# The Rare Togetherness of Bladder Leiomyoma and Neurofibromatosis

**DOI:** 10.1155/2018/2302918

**Published:** 2018-01-08

**Authors:** Cem Yucel, Salih Budak, Erdem Kisa, Orcun Celik, Zafer Kozacioglu

**Affiliations:** Department of Urology, Tepecik Training and Research Hospital, Izmir, Turkey

## Abstract

Neurofibromatosis Type 1 (Von Recklinghausen disease) is a common, autosomal dominant hereditary disorder characterized by involvement of multiple tissues derived from the neural crest. Urinary system involvement in neurofibromatosis is a rare condition. Leiomyoma of the bladder is a rare benign mesenchymal tumor. In this case, our experience and approach regarding the bladder leiomyoma development in a patient diagnosed with neurofibromatosis are presented and the literature data has been reviewed.

## 1. Introduction

Neurofibromatosis Type 1 also called Von Recklinghausen disease is one of the most common neurogenetic diseases. Rate of incidence in the world is 1/3500 [1]. It is an autosomal dominant disease characterized by multisystemic lesions. It has been proved that the gene causing the disease is located on the chromosome 17 [2]. Urinary system involvement in neurofibromatosis is a rare condition. The most frequently affected organ in the urinary system is bladder. Leiomyoma is a rare mesenchymal tumor of the bladder. Histopathologically, leiomyoma of the urinary bladder is composed of fascicles of smooth muscle fibres that are separated by connective tissues. They are noninfiltrative smooth muscle benign tumors with no mitotic activity, cellular atypia, or necrosis. Benign mesothelial tumors of the bladder account for 1–5% of all bladder tumors [3]. A case of leiomyoma that is a rare benign tumor developing in the bladder of the patient with neurofibromatosis is reported in this study.

## 2. Case Report

**Figure 1:**
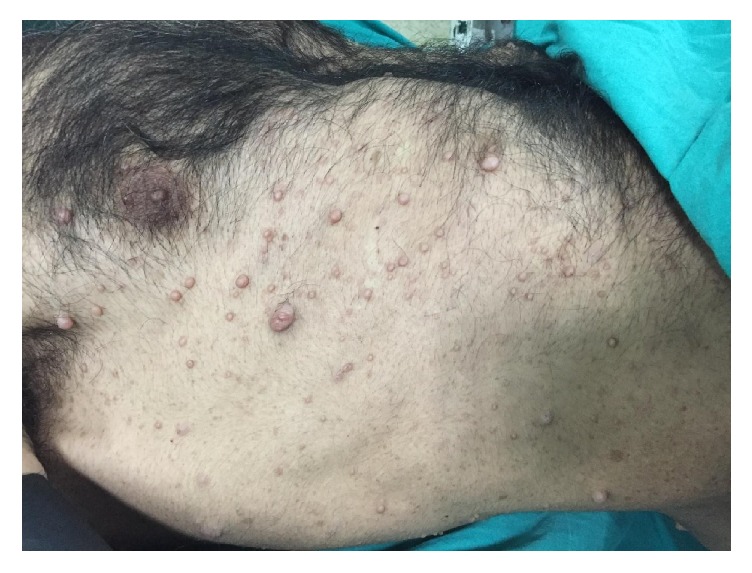
Neurofibromas and cafe au lait spots on the skin.

**Figure 2:**
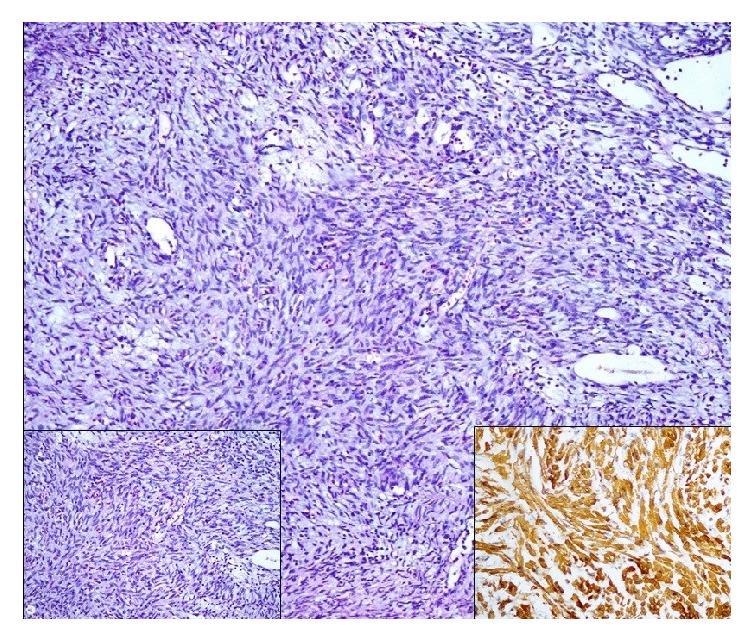
Spindle cell lesion in resection specimen (H&E stain, 100x). No atypia, left inset (H&E stain, 200x). Positive expression with smooth muscle actin immunohistochemistry, right inset (100x).

**Figure 3:**
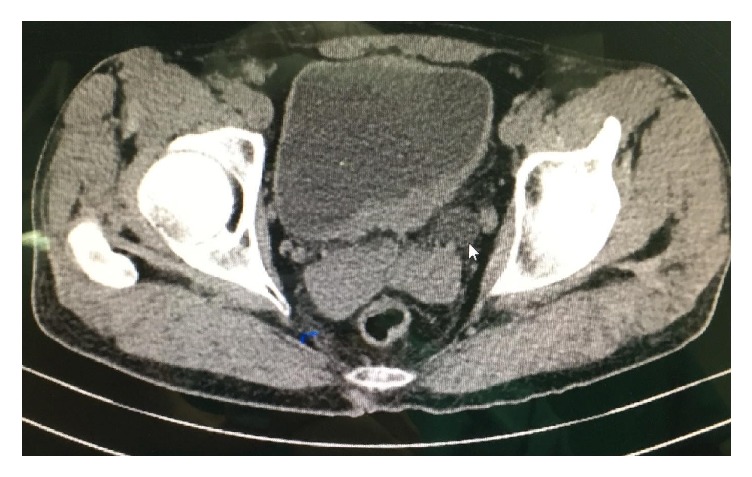
Mass on the left wall of bladder and ureter dilatation. The arrow shows the dilatation of ureter.

A 44-year-old man was admitted to our institution in May 2016 complaining of suprapubic pain. He had a history of brown spots and freckles on his body since he was a child. We detected common skin nodules on his whole body, particularly on his torso in physical examination (Figure 1). Two years ago, in neurosurgery department brainstem glioma was detected with magnetic resonance imaging because of presenting of headache and follow-up program was applied. The patient diagnosed with neurofibromatosis had no hematuria symptom and underwent an abdominal ultrasonography (USG). USG of the abdomen had revealed 18 × 22 mm a well-circumscribed mass lesion in the inferior bladder wall. He underwent the transurethral resection of the bladder (TURB). Pathological result of the TURB was leiomyoma of the bladder. In histopathology, there was no mitosis and atypia. In addition, there was a proliferation of spindle-shaped cells, in addition to an eosinophilic cytoplasm and fibres with haematoxylin and eosin stain. Immunohistochemistry was positive for smooth muscle actin (SMA) and vimentin (Figure 2). The patient was included in the follow-up program. Abdominal computed tomography (CT) applied to the patient because of his left side and back pain in third-month visit. CT revealed grade 3 left hydroureteronephrosis and a tumor formation of 6 × 3.5 cm extending from the left lateral wall to the anterior wall of the bladder (Figure 3). Reoperation was applied to the patient. A solid polypoid mass blocking left orifice and covered by the normal bladder mucosa was detected on the left lateral wall in the operation. The performed resection failed to visualize the left orifice. The results of the samples sent to the pathology laboratory were reported as mesenchymal tumor formation. We recommended nephrostomy for protecting the function of kidney and partial cystectomy for treatment but the patient rejected all treatment options.

## 3. Discussion

Neurofibromatosis is a common neurogenetic disease. Neurofibromatosis was first described by Von Recklinghausen and the diagnosis requires the presence of at least two of the clinical symptoms in the Table 1 [4]. Leiomyoma is a mesenchymal benign tumor of the bladder that is rare [5]. Bladder wall involvement of neurofibromatosis is rare and there are about 60 case reports in the literature. Leiomyoma development in neurofibromatosis is observed mainly in the gastrointestinal system. Only one case of bladder leiomyoma development in patient diagnosed with neurofibromatosis has been reported until today in the literature [6]. The study with the largest patient population regarding the bladder leiomyoma was performed by Goluboff et al., in which 37 cases of bladder leiomyoma were evaluated [7]. In their study, there were obstructive symptoms in 49%, irritative symptoms in 38%, hematuria in 11%, and lumbar pain in 13% of the patients. On the other side, 19% of the patients were reported as asymptomatic. The masses in 57% of the patients were palpable during the bimanual examination. Their study also showed that leiomyoma is mostly seen in women, especially on the third and fourth decades of life.

Bladder leiomyoma can be diagnosed by ultrasound, tomography, and cystoscopy. Submucosal leiomyoma may be pedunculated or polypoid and cause irritative symptoms, suprapubic pain, urethral obstruction, lower urinary tract infection, and hematuria. Patients with intramural leiomyoma present with pelvic mass and obstructive symptoms are notable. Extramural leiomyoma may reach large sizes and these patients present with the symptoms according to mass compression [8]. In this case, leiomyoma was submucosal and observed with the complaint of suprapubic pain. Hematuria was not detected in the patient.

Treatment of bladder leiomyoma is transurethral resection; however, large tumors can be treated with open partial cystectomy if the tumor location is appropriate. In our case, diagnosis was confirmed by ultrasound and tomography, and transurethral resection was used for the treatment.

## Figures and Tables

**Table 1:** Neurofibromatosis Type 1 diagnostic criteria.

(1) Six or more pigmentations greater than 5 mm before puberty and greater than 15 mm after puberty (cafe au lait)
(2) One plexiform neurofibroma or more than two neurofibromas of any type
(3) Axillary or inguinal freckling
(4) Optic glioma
(5) Two or more “Lisch” nodules (iris hamartoma)
(6) Bone lesions
(7) Presence of at least one of these clinical findings in first-degree relatives
